# Spontaneous Coronary Artery Dissection-Induced Takotsubo Syndrome

**DOI:** 10.7759/cureus.24945

**Published:** 2022-05-12

**Authors:** Roshni O Prakash, Teja S Chakrala, Steven M Brady, Sahil Prasada, Ellen C Keeley

**Affiliations:** 1 Department of Medicine, University of Florida Health, Gainesville, USA; 2 Department of Medicine and Department of Cardiology, University of Florida Health, Gainesville, USA; 3 Department of Medicine and Department of Cardiovascular Medicine, University of Florida Health, Gainesville, USA

**Keywords:** left sided catheterization, risk factor, chest pain, cardiomyopathy, coronary angiography, cardiovascular disease

## Abstract

A case of an anxious 59-year-old woman, who presented with chest pressure, nausea, and vomiting, is described. After hours of symptoms that worsened despite medical management, cardiac catheterization was performed. Angiography revealed diffuse, long, tubular disease of multiple coronary vessels. Additionally, left ventriculography was consistent with Takotsubo syndrome. Based on both coronary angiography and left ventriculography, it was determined that this patient had concomitant spontaneous coronary artery dissection and Takotsubo syndrome.

## Introduction

Spontaneous coronary artery dissection (SCAD) has emerged as a rare cause of acute coronary syndrome, predominantly among women [1, 2]. It is proposed that an intimal flap or intramural hematoma forms as layers of the coronary artery spontaneously separate. SCAD has been associated with patients with fibromuscular dysplasia, inflammatory bowel disease, pregnancy, and patients experiencing extreme emotional stress or physical exertion [3, 4]. The Takotsubo syndrome (TTS) is another condition that shares many features with SCAD. Both SCAD and TTS frequently present with signs and symptoms of acute coronary syndrome [5]. TTS is characterized by transient ventricular dysfunction in the absence of significant coronary artery disease [5]. Recent studies have discussed the causal links between SCAD and TTS, noting that SCAD may serve as a physical trigger for the development of TTS [5].

We report a case of a woman presenting with cardiovascular symptoms preceded by emotional stress. She was found to have both SCAD and TTS based on angiography. Our case demonstrates the importance of considering the simultaneous presence of these diagnoses as well as a causal relationship between SCAD and TTS.

## Case presentation

A 59-year-old woman from Ohio was in Florida visiting family when she developed exertional chest pressure, nausea, and vomiting. Initially, she thought it was due to swallowing ocean water during a beach trip. After two days of intermittent symptoms, she developed left arm numbness which prompted her to go to the emergency department for evaluation. 

The patient had a history of generalized anxiety disorder (GAD), major depressive disorder (MDD), and chronic back pain with a history of lumbar disc herniation. She had no known cardiac history. Family history was significant for myocardial infarction in her mother. She endorsed a five-pack-year smoking history.

Upon admission, the differential diagnosis included gastroesophageal reflux disease, esophageal spasm, myocardial ischemia or infarction, unstable angina, myocarditis, pulmonary embolism, SCAD, ischemic cardiomyopathy, and coronary artery vasospasm. 

**Figure 1:**
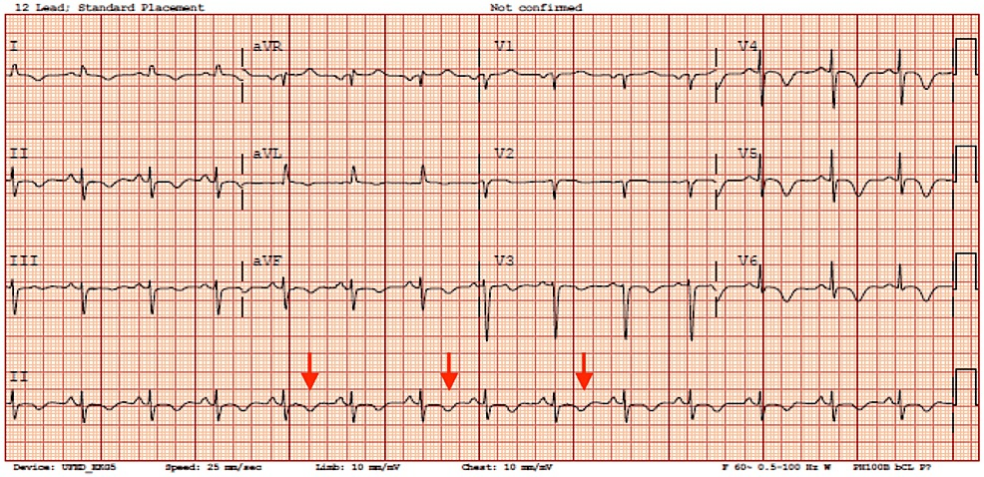
12-lead electrocardiogram showing T-wave inversions in the anterior leads.

On presentation, the patient was afebrile, blood pressure 131/98 mmHg, heart rate 102 bpm, respiratory rate 30 breaths/min, and SpO2 was 93% on room air. There was mild tenderness to palpation at the midepigastric region, but the rest of her exam was unremarkable. Laboratory results were significant for lactic acid level of 3.8 mmol/L and high sensitivity troponin I level of 1,655 pg/mL. A 12-lead electrocardiogram showed diffuse T-wave inversions (Figure 1).

She was started on IV heparin and nitroglycerin. A cardiac CT angiogram showed multifocal, moderate to severe plaques in the left anterior descending (LAD) coronary artery and to a lesser degree in the right coronary artery. It also showed moderately reduced left ventricular systolic function with akinetic apical and mid segments. Her calcium score was 4.6. Due to continued chest pain and rising troponin-I levels, she was taken urgently to the cardiac catheterization lab.

**Figure 2:**
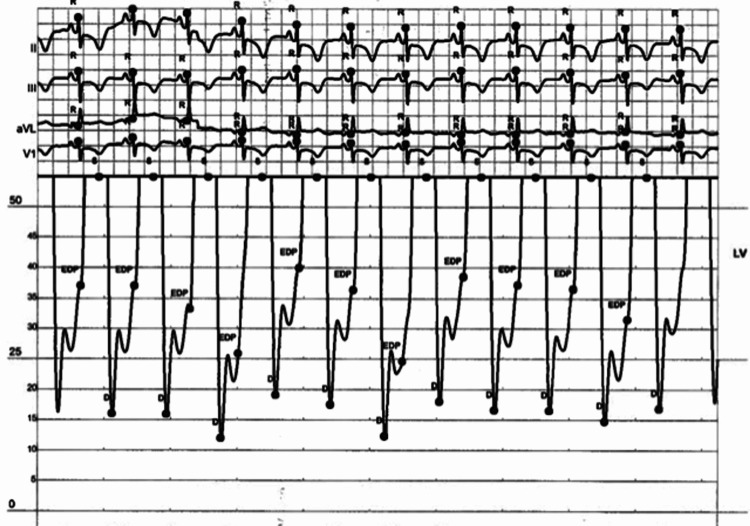
Left ventricular end-diastolic pressure tracing.

**Figure 3:**
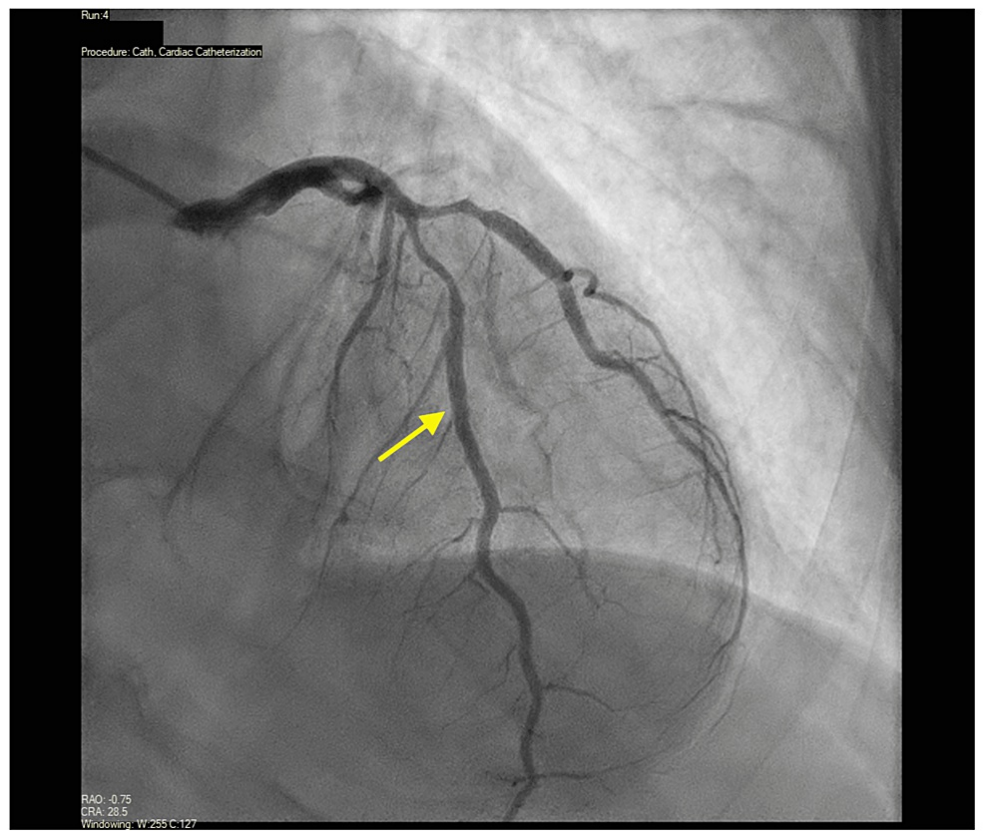
30º anterior-posterior view of the left anterior descending artery and its diagonal branch.

**Figure 4:**
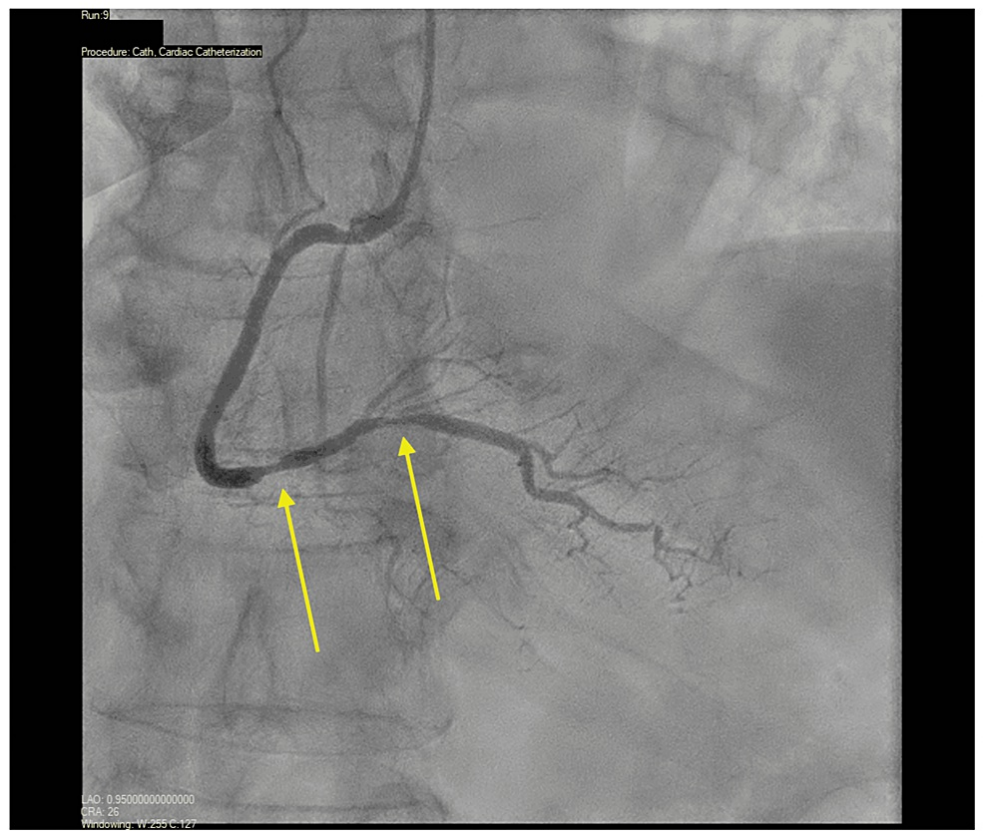
30º anterior-posterior view of the right coronary artery.

In the laboratory, left ventricular end-diastolic pressure was elevated (35 mmHg) (Figure 2). Coronary angiography revealed diffuse, long, tubular, non-obstructive disease of the LAD, its diagonal branch (Figure 3) and the right coronary artery​​ (Figure 4). Invasive physiologic evaluation using fractional flow reserve confirmed non-flow limiting lesions of the LAD (0.91) and its diagonal branch (0.94). Left ventriculography showed evidence of apical ballooning (Video 1). Coronary angiography and left ventriculography findings were consistent with concomitant SCAD and TTS, respectively. 

**Video 1:** 30º right anterior oblique left ventriculogram showing apical ballooning.

After the procedure, she was started on low dose aspirin, high-intensity statin, and goal-directed medical therapy including a beta-blocker and an angiotensin-converting enzyme inhibitor. High sensitivity troponin I peaked at 1,802 pg/mL. After 24 hours, the patient became pain-free and IV nitroglycerin was discontinued. Her high sensitivity troponin I was finalized at 731 pg/mL. Additional studies performed during her hospitalization included carotid and renal duplex studies which were unremarkable. 

## Discussion

Spontaneous coronary artery dissection and TTS are phenomena predominantly found in women and known to be triggered by emotional and physical stressors [1, 2]. Other risk factors for SCAD include fibromuscular dysplasia, systemic inflammation, connective tissue disease, and pregnancy. Presentations of SCAD range from mild chest pain to sudden cardiac death, with the most frequent presentation being that of myocardial infarction [1-3]. The presentation of TTS, however, includes substernal chest pain, dyspnea, syncope, arrhythmias, and symptoms of heart failure [3]. 

Spontaneous coronary artery dissection is an overarching term with three subtypes as elucidated in the Yip-Saw classification: Type 1 demonstrates multiple lumens on angiography, Type 2 involves extensive mid and distal arterial segments, and Type 3 mimics atherosclerosis and can be challenging to distinguish without further imaging [4]. Type 2 SCAD is characterized by diffuse smooth and long lesions (>20mm) on angiography with abrupt changes in vessel diameter, classic findings seen in our patient (Figures 3, 4). Although optical coherence tomography and intravascular ultrasound may be performed if the diagnosis of SCAD is uncertain, up to 8% of patients with SCAD have been reported to experience procedural complications from intravascular imaging [1]. Risks include propagation of the existing dissection, a procedurally induced dissection, and decreased blood flow after imaging, and should therefore only be pursued in cases of uncertainty [1-3]. 

Due to overlapping risk factors and presentations, simultaneous development of TTS and SCAD is more common than previously thought [3, 5]. Several theories have been hypothesized on the relation and underlying pathogenesis of TTS and SCAD, suggesting a common origin. In our patient, we think that SCAD led to an acute ischemic insult inducing the stress cardiomyopathy. This has been theorized to occur in prior associations as well suggesting that excessive left ventricular basilar contraction in TTS with the akinetic apical systolic ballooning and neighboring segments may act as a substrate for the development of SCAD in a predisposed individual [5]. An alternate explanation is that chest pain experienced by predisposed patients with ongoing SCAD may serve as a physical trigger for TTS [3, 5]. 

Like in TTS, a history of psychiatric conditions has been found to be a major risk factor in the development of SCAD. Few reported cases point to the importance of focusing on the possible association between conditions such as GAD and MDD and concurrent SCAD-TTS cases [6, 7]. Our case contributes to this literature as our patient not only had a history of anxiety and depression but also reported experiencing multiple stressors prior to symptom onset. A widely postulated mechanism behind this association relates to a surge in catecholamine levels in the setting of emotional distress, followed by an increase in coronary artery vasospastic activity [8]. A rise in arterial wall stress subsequently follows, leading to intimal rupture and disruption of the vaso-vasorum [8]. Dedicated SCAD cardiac rehabilitation programs, with incorporated psychosocial counseling techniques, have been trialed with observed improvements in long-term cardiovascular outcomes in these patients [8]. 

Spontaneous coronary artery dissection is often underdiagnosed as these patients frequently have concurrent atherosclerosis. When a lesion is identified during angiography and deemed the culprit, further testing (including left ventriculography or transthoracic echocardiography) may not always be performed [1, 4, 9]. Thus, the diagnosis of TTS could be missed. It is important for healthcare providers to be aware of both presentations to prevent patients from undergoing unnecessary therapeutic interventions. An understanding of the association between SCAD and TTS, which is more common than previously regarded, is crucial in helping to make correct diagnoses. The key findings discussed in a published case report include SCAD in the diagonal artery, SCAD leading to myocardial infarction, and induced myocardial stunning with left ventricular mid-apical ballooning [8]. In a registry, episodes were reported of multivessel SCAD with concurrent TTS, which was precisely what was found in our patient [3]. Our case involved both the LAD and the RCA, making it a diagnosis of multivessel SCAD. Typically, treatment of SCAD is conservative with a small proportion of patients requiring revascularization therapies [2, 4]. Recurrent events are frequent in both patients with SCAD and TTS and therefore require close follow up [2, 4, 6, 8, 10]. 

Our patient was referred to a SCAD specialist in her home state upon discharge. 

## Conclusions

SCAD is underdiagnosed and associated with significant morbidity and mortality, requiring proper recognition and treatment. Due to the paucity of cases reported, along with the diversity of clinical pictures and angiographic patterns at the time of presentation, this condition is particularly prone to delayed or inaccurate diagnoses. We describe a case of concomitant Type 2 SCAD and TTS, suggesting a common pathogenetic mechanism. Awareness of the concomitant occurrence of these two entities is paramount to facilitating appropriate management of affected patients. 
